# Changes in Insurance Physicians’ Attitudes, Self-Efficacy, Intention, and Knowledge and Skills Regarding the Guidelines for Depression, Following an Implementation Strategy

**DOI:** 10.1007/s10926-012-9378-9

**Published:** 2012-07-05

**Authors:** Feico Zwerver, Antonius J. M. Schellart, Johannes R. Anema, Allard J. van der Beek

**Affiliations:** 1 Department of Public and Occupational Health, EMGO Institute for Health and Care Research, VU University Medical Center, Amsterdam, The Netherlands; 2 Research Center for Insurance Medicine, Amsterdam, The Netherlands; 3 Dutch National Institute for Employee Benefits Schemes, Amsterdam, The Netherlands

**Keywords:** Guideline adherence, Insurance physicians, Guidelines for depression, ASE determinants

## Abstract

*Introduction* To improve guideline adherence by insurance physicians (IPs), an implementation strategy was developed and investigated in a randomized controlled trial. This implementation strategy involved a multifaceted training programme for a group of IPs in applying the guidelines for depression. In this study we report the impact of the implementation strategy on the physicians’ attitude, intention, self-efficacy, and knowledge and skills as behavioural determinants of guideline adherence. Any links between these self-reported behavioural determinants and levels of guideline adherence were also determined. *Methods* Just before and 3 months after the implementation of the multifaceted training, a questionnaire designed to measure behavioural determinants on the basis of the ASE (attitude, social norm, self-efficacy) model was completed by the intervention (n = 21) and the control group (n = 19). Items of the questionnaire were grouped to form scales of ASE determinants. Internal consistency of the scales was calculated using Cronbach’s alphas. Differences between groups concerning changes in ASE determinants, and the association of these changes with improvements in guideline adherence, were analyzed using analysis of covariance. *Results* The internal consistency of the scales of ASE determinants proved to be sufficiently reliable, with Cronbach’s alphas of at least 0.70. At follow-up after 3 months, the IPs given the implementation strategy showed significant improvement over the IPs in the control group for all ASE determinants investigated. Changes in knowledge and skills were only weakly associated with improvements in guideline adherence. *Conclusions* The implementation strategy developed for insurance physicians can increase their attitude, intention, self-efficacy, and knowledge and skills when applying the guidelines for depression. These changes in behavioural determinants might indicate positive changes in IPs’ behaviour towards the use of the guidelines for depression. However, only changes in knowledge and skills related to the use of the guidelines were associated with improvements in IPs’ actual performance when applying the guidelines.

**Electronic supplementary material:**

The online version of this article (doi:10.1007/s10926-012-9378-9) contains supplementary material, which is available to authorized users.

## Introduction

Health care guidelines are intended to incorporate evidence-based medicine into the daily practice of physicians [1–3]. Encouraging the use of guidelines in daily practice is important for improving uniformity and quality in health care. However, the implementation of guidelines is a complex process influenced by many factors, such as the behaviour of the physicians, the guidelines themselves or the way in which the guidelines are implemented [4, 5]. There are numerous possible barriers in this process, which range from the distribution of guidelines to the use of guidelines in practice by physicians. Such barriers can be external, such as lack of availability, lack of practical relevance of the guidelines, or lack of support by the organization. But barriers can also be internal, for example lack of familiarity with the guidelines, lack of physicians’ agreement with guidelines, negative attitudes in general towards guidelines (some physicians refer to guidelines as ‘cookbook medicine’), or lack of self-efficacy in using guidelines [5].

Physicians’ behaviour towards using guidelines may well be one such barrier, and therefore requires investigation. Physicians give various reasons for their reluctance to use guidelines and these reasons include the following: guidelines do not suit the individual problems of their patients, using guidelines does not improve their work (so-called lack of outcome expectancy), using guidelines limits their professional independency, or there is no pressure from patients or staff to use guidelines. The challenge for educational interventions is therefore to positively influence physicians’ behaviour towards the use of guidelines.

Educational programmes for physicians—as part of a guideline implementation strategy—have been evaluated in other studies and have produced varying results. Changing physicians’ behaviour, such as increasing their guideline adherence, is possible, but such change requires comprehensive approaches at different levels, tailored to specific settings and persons [6]. With regard to the educational aspects of physicians’ guideline adherence, the strongest effects can be expected from multifaceted interventions rather than more formal types of education such as stand-alone lectures [7].

In 2005, the Dutch Health Council implemented guidelines in the field of insurance medicine [8]. For one of these guidelines—the guidelines for depression—we subsequently set up a research project to evaluate a newly developed implementation strategy [9]. This implementation strategy consisted of a tailor-made training programme for insurance physicians (IPs) in which—facilitated with various tools—they learned to apply the guidelines for depression. The information in all tools was evidence-based, derived from the guidelines, and readily applicable in practice.

**Fig. 1:**
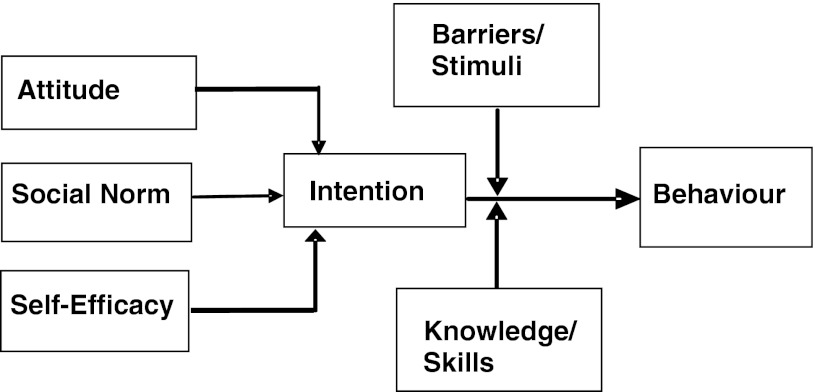
ASE-model [11]

Explanations for physicians’ behaviour with regard to guideline adherence can be found in the Attitude, Social Norm, Self-Efficacy model (ASE model), which is derived from the Theory of Planned Behaviour (TPB) [10, 11]. The TPB is a theory designed to predict and explain human behaviour in specific contexts. Behaviour (applying guidelines) is influenced by intentions to perform that behaviour. In turn, these intentions are preceded by attitude, social norm and self-efficacy with regard to the desired behaviour. IPs are thought to have a certain attitude (positive or negative) towards guidelines that influences their intention to use them. Furthermore, IPs’ intention to use guidelines could be determined by their colleagues (social norm) or by their perception of behavioural control, i.e. the degree to which they feel comfortable using guidelines (self-efficacy). The relationships between the determinants of behaviour—such as attitude, social norm, self-efficacy and intention—and the interfering stimuli or barriers involved in performing expected behaviour are shown in the ASE model (Fig. 1).

We started by investigating and describing the behaviour of IPs on the basis of the ASE model by means of a cross-sectional study at baseline [12]. In this study we investigated the relationships between the IPs’ intention to use the guidelines for depression and their self-reported determinants of behaviour towards their use of the guidelines. However, the ASE model could only partly be confirmed. We found no relationship between intention and use of the guidelines while it is this relationship which represents the main line of the ASE model. We did however find determinants of behaviour that influenced the IPs’ intention to use the guidelines, namely the influence of colleagues, self-efficacy and the way in which guidelines are implemented.

We subsequently carried out a randomized controlled trial (RCT) to evaluate the implementation strategy that we developed for the guidelines for depression, with guideline adherence by IPs as primary outcome [13]. This RCT showed an improvement in guideline adherence for the IPs given the implementation strategy.

Considering the limited findings with regard to the ASE model at baseline, but given the clear results of the RCT, we wondered what differences and changes in ASE determinants might occur between the groups of the RCT as a result of the implementation strategy. The implementation strategy was predominantly aimed at training the IPs’ skills and improving their self-efficacy by giving them practice in applying the guidelines with the help of tools, and in analyzing case studies of clients with depression played by actors presented on video. If the aim is to change behaviour, improving knowledge of the guidelines alone might not be sufficient. If so, it is important to know whether these differences or changes in ASE determinants could be related to the improvements observed in physicians’ guideline adherence, and, above all, which of the ASE determinants might predict improvements in physicians’ guideline adherence.

The aims of the present study are firstly to describe changes in determinants of IPs’ behaviour following an implementation strategy for the guidelines of depression and secondly to investigate their association with the changes observed in guideline adherence.

The Medical Ethics Committee of VU University Medical Center approved the study design and the Netherlands Trial Registration accepted the RCT: NTR1863.

## Methods

### Participants

The participants were insurance physicians (IPs) that we recruited with the help of the Netherlands School of Public and Occupational Health (NSPOH) and who attended a post-graduate course in applying the insurance medicine guidelines for depression. The IPs participated on a voluntary basis. The RCT was integrated into this course. Inclusion criteria for enrolment of participants in the RCT was that they were (1) either registered as an IP, or still following the post-graduate course in Insurance Medicine, and (2) conducting disability assessments of clients under commission of the Dutch National Institute for Employee Benefits. In the Netherlands, the Institute for Employee Benefits (the Institute) is responsible for evaluating disability claims. Dutch employees (known by the Institute as clients) can claim disability benefits after having been sick-listed for 104 weeks, during which time the employees are treated by occupational physicians. After 104 weeks the employees are transferred from the occupational physician to the IP, who assesses the work disability claim at the Institute. The level of the employee’s benefit is then determined on the basis of this work disability assessment by an IP. An IP is a physician who has completed a four-year post-graduate course combined with practice as an IP, resulting in registration as an IP.

Forty-three IPs applied for the training on a voluntary basis. All 43 were included and they were individually allocated in order of registration either to the intervention group or to the control group using a random-sequence table. To prevent unequal allocation across groups, the participants were stratified before randomization according to three prognostic factors: age, gender and registration as an IP. A research assistant performed the randomization and stratification procedure. The participants were all blinded for the design of the RCT and were given badges with a unique number, which was also written on their study materials. This meant that the researchers were also blinded for their group allocation. By the time it was clear whether participants were in the intervention or control group, due to the kind of training they received, the baseline measurements of the RCT had already been completed. The group of IPs was representative of the approximately 900 IPs working at the Institute [14]. The training programme was located at the NSPOH in Amsterdam. The participants went there on four different days from March to June 2009 for the measurements, the intervention programme and the control programme. Meanwhile they worked as practising IPs at the Institute.

### Study Design

We conducted an RCT that compared an intervention group (IG) and a control group (CG) for guideline adherence and ASE determinants. Participants in the intervention group received the implementation strategy developed for the guidelines for depression. The control group received an alternative programme that did not interfere with the intervention programme. After completion of the RCT measurements, both groups followed the remaining training programme. Objective measurements regarding guideline adherence were carried out using performance indicators (PIs). The development and reliability of these PIs have been reported previously [15], as have the results of the RCT based on the PI scores observed [13]. The minimum sample size required to detect a change in the primary outcome of the RCT—guideline adherence—was determined by means of power analysis [13].

Another part of the same RCT measured ASE determinants using two questionnaires, one before the intervention and the other after the intervention. The ASE determinant ‘social norm’ was left out of this study, because changes in social norm are beyond the scope of the intervention.

### Questionnaires

Questionnaire constructs for measuring the four determinants attitude, self-efficacy, intention, and knowledge and skills were developed from the concepts in the ASE model [11]. The questionnaires at baseline (T0) and at follow-up after 3 months (T1) both contained identical constructs for the ASE determinants (attitude, self-efficacy, knowledge, and intention). The questionnaires used 38 items with responses on a five-point scale ranging from ‘strongly disagree’ to ‘strongly agree’. The items were clustered in four scales for the constructs determined by the ASE determinants (see Appendix in ESM for the questionnaires).

### Intervention

The implementation strategy was developed with the help of users and experts. It was aimed at improving the availability and the practicability of the guidelines for depression. The intervention consisted of a multifaceted training programme for IPs in applying the guidelines for depression. The different components of the programme included interactive presentations by experts and exercises in subgroups, where IPs practised assessing clients with depression played by actors on video. The IPs’ trainers provided them with feedback. Individual assignments for IPs involved practice in writing disability reports following the feedback from the IP trainers. A number of evidence-based tools (a plastic desk mat listing a summary of the guidelines, as well as two different guidelines checklists and the Hamilton Rating Scales for Depression [16]) were developed for this programme, aimed at improving the applicability of the guidelines. The IPs were instructed how to use these tools. Learning objectives for the IPs were to use the tools to improve their diagnostic skills, to improve their assessment of work ability of clients with depression, and to write down their findings and conclusions in well-argued reports. The objective for the IPs’ reports was that they should be more transparent and more evidence-based and should contain well-argued assessments of a client’s work ability. Two of the authors (FZ and JRA) acted as the two IP trainers in the intervention program.

### Control Group

For reasons of recruitment and equal treatment, the control group acted as a waiting list control and was later given the same educational programme as the intervention group. At the same time the intervention group received the intervention programme, the control group participated in a ‘placebo training’, which was a programme in motivational interviewing. The content of the motivational interviewing programme did not interfere with the intervention or the guidelines for depression, because these two programmes shared no common ground.

### Data Collection and Outcome Measures

Data were collected using two questionnaires at baseline (T0) and at follow-up after 3 months (T1). The first questionnaire included items for the baseline characteristics of the participating IPs. The questionnaires were filled in and collected while participants attended the course. The primary outcomes were the IPs’ behavioural changes (T1 vs. T0) towards the guidelines, expressed in terms of ASE determinants. We also determined the association, if any, between these self-reported ASE determinants and the main outcome of the RCT, i.e. the IPs’ levels of guideline adherence expressed in terms of a performance indicator (PI) sum score.

### Statistical Analysis

The RCT required equal allocation to both groups of the participant characteristics age, gender and registration as an IP. If necessary, we corrected for confounding variables in the analyses performed. Both questionnaires contained items that formed constructs representing the four scales of the ASE determinants. Scale scores were obtained by adding the responses to the items within each scale. The internal consistency within each scale was determined with Cronbach’s alphas. A Cronbach’s alpha of 0.60 at baseline was considered to be the minimum for consistency. To investigate the differences between the groups due to the intervention, a one-way analysis of covariance (ANCOVA) was conducted (*p* < 0.05). The group was the independent variable, the ASE determinant at baseline (T0) was the covariate, and the ASE determinant at follow-up (T1) was the dependent variable. All dependent variables were normally distributed, and the homogeneity-of-slopes assumption was tested for these variables—both conditions for the valid use of ANCOVA analysis. To address the second aim of this study, we used an ANCOVA model for change [17] with as dependent variable the PI sum score at T1. As independent variables we used as factor the intervention group and control group; and as covariates we used the PI sum score at T0, the relevant ASE determinant at T0, and the interaction between the change of the same ASE determinant at T1 and the group. We were especially interested in this interaction effect.

In all ANCOVA analyses we corrected for a confounding variable seen in the participating IPs: there was a significant difference between the intervention group and control group in the mean number of clients with depression that they assessed for work disability each month.

The statistical analyses were performed at the individual level of the participants in the RCT according to the per-protocol principle and using SPSS version 15.0 (SPSS, Chicago, Illinois; 2006). Because the trial in this study was an efficacy trial, in which we were interested in knowing whether the intervention works for a group of IPs in a specific controlled setting, rather than an effectiveness study carried out in real practice, we chose to present the figures of the per-protocol analyses. We also performed an intention to treat analysis, thereby including the three IPs lost to follow up (21 in the intervention group, and 22 in the control group), but this had no influence on the results.

## Results

A total of 43 IPs applied to take part in the post-graduate course: 21 received the intervention programme while 19 were in the control group. Three IPs were lost to follow-up. The response rate of the questionnaires was 100 %. The baseline characteristics are summarized in Table 1. The IPs’ behaviour regarding the guidelines for depression as determined by the ASE variables, which was the primary outcome measure, was related to the mean number of clients with depression per month assessed by the participating IPs.

**Table 1:** Baseline characteristics of the participants of the post-graduate course

	Intervention (n = 21)	Control (n = 19)	*p* value*
Years of age	51.1 (6.2)	50.5 (6.7)	0.92
Working hours/week	31.1 (9.2)	31.8 (9.9)	0.82
Years of experience as IP	15.6 (7.9)	15.4 (8.1)	0.92
Mean number of clients with depression per month	5.3 (3.7)	9.3 (5.6)	**0.01**
Gender M/F (%)	52.3/47.7	47.3/52.7	0.75
Being registered as IP (%)	85.7	84.2	0.89

Means and standard deviations are given for continues variables. Percentages are given for the categorical variables

* *p* value of the independent *t* test and *p* value of the Chi-squared, respectively, between the intervention and the control group; significant differences are bold

### Reliability

For each of the self-reported scales used for the ASE determinants, Cronbach’s alphas were calculated to test their internal consistency. All ASE determinants had a Cronbach’s alpha of at least 0.70, which indicates that the self-reported measures utilized in this study were sufficiently reliable (see Table 2).

**Table 2:** Internal consistency analysis (n = 40)

Variables based on determinants of the ASE-model	Items	Cronbach’s alpha Baseline (T0)	Cronbach’s alpha Follow-up (T1)
Attitude (GD)	9	0.77	0.77
Self-efficacy	11	0.75	0.86
Knowledge and skills	8	0.77	0.72
Intention	10	0.75	0.79

*GD* guidelines for depression

### Outcomes

Table 3 shows, for each of the ASE variables, the means, the standard deviations at baseline and at follow-up, and the *p* values of the between-group difference at follow-up, corrected for baseline values and for the confounding variable ‘mean number of clients with depression assessed per IP per month’ (*p* < 0.05). The intervention had a significant effect on all the ASE variables investigated. At follow-up the participants of the intervention group had not only a more positive self-reported attitude, self-efficacy and intention towards the guidelines for depression than the participants in the control group, they also showed an improvement in knowledge and skills for applying the guidelines. In the intervention group, attitude and intention both improved by 12 %, self-efficacy by 10 %, and knowledge and skills by 5 %. In the control group, attitude and intention stayed almost equal, while self-efficacy and knowledge and skills even decreased by 9 and 15 %, respectively.

**Table 3:** Mean scores (SD) on the ASE-variable scales and *p* values of the differences between intervention group (IG) and control group (CG) at follow-up (T1)

ASE-variables (scale)	Intervention (n = 21)		Control (n = 19)		*p* value^a^
	T0	T1	T0	T1	
Attitude (scale 9–45)	33.9 (6.2)	38.1 (4.7)	31.8 (4.1)	33.7 (3.2)	**0.003**
Self-efficacy (scale 11–55)	35.8 (5.1)	39.5 (5.5)	34.6 (5.9)	31.6 (4.9)	**0.000**
Knowledge and skills (scale 8–40)	27.9 (5.4)	29.3 (4.5)	27.7 (5.5)	23.3 (4.0)	**0.000**
Intention (scale 10–50)	34.9 (6.0)	39.2 (5.8)	34.5 (5.5)	34.4 (4.6)	**0.014**

^a^Ancova analysis: *p* values of differences between control group and intervention group at follow-up, corrected for baseline value and the confounding variable (number of clients with depression). Bold figures are significant

The ANCOVA analyses for the second aim of this study showed no significant interaction effects between changes in ASE determinants at T1 and group for the guideline adherence observed at T1 (see Table 4). With regard to the ASE determinants, only the interaction effect of the changes in knowledge and skills showed a weak association (*p* = 0.093) with the improvement in guideline adherence observed at T1. At the group level, only the associations for the control group tended to be weakly significant and negative for self-efficacy (*p* = 0.111) as well as for knowledge and skills (*p* = 0.078). The changes in attitude and in intention were not related to the improvement in guideline adherence observed at T1 (*p* = 0.950 and *p* = 0.741, respectively).

**Table 4:** Associations between observed guideline adherence at T1 (PI sum scores) with change of ASE-variables (self-reported) atT1 regarding the guidelines depression for intervention group (IG, n = 21) and control group (CG, n = 19)^a^

ASE-variables	Parameter	*p* value
Group*Dattitude T1		0.950
Group = CG	0.006	0.991
Group = IG	0.147	0.758
Group*Dself-efficacy T1		0.213
Group = CG	−0.578	0.111
Group = IG	0.081	0.758
Group*Dknowledge and Skills T1		0.093
Group = CG	−0.672	0.078
Group = IG	0.114	0.801
Group*Dintention T1		0.741
Group = CG	0.001	0.996
Group = IG	−0.258	0.455

^a^Ancova analysis: dependent variable dependent variable = PI sum score at T1; D = difference of ASE Variable T1–T0; parameter estimates and their *p* values, with group as factor and corrected for baseline values of PI sum score and of the concerning ASE-variable, and for the confounding variable (number of clients with depression)

## Discussion

### Main Results

In this study we investigated the effect of a newly developed implementation strategy on insurance physicians’ attitude, self-efficacy, intention, and knowledge and skills towards the guidelines for depression. After 3 months, IPs who participated in the training course demonstrated a more positive attitude to the guidelines for depression, a higher intention to use them, more self-efficacy, and more knowledge and skills in applying the guidelines for depression than their colleagues in the control group.

### Interpretation

Our results show that the implementation strategy had the most impact on the physician’s attitude, self-efficacy, and their intention to apply the guidelines and less impact on the physician′s knowledge and skills. According to the ASE model, attitude and self-efficacy are the precursors of intention, which in turn predicts behaviour, in this case the physicians’ guideline adherence. Physicians who were confident about applying the guidelines, and who had a positive attitude, showed a higher intention to use the guidelines. If attitude, self-efficacy and intention increase, subsequently facilitated by knowledge and skills, then behaviour should change positively. Whether this is a clinically relevant change should be studied in real practice on patient outcomes. This change might also be clinically relevant, because if IPs are more inclined to apply guidelines in practice, their work disability assessments might be more evidence-based, and their assessments might be executed more uniformly. The implementation strategy indeed resulted in a change of behaviour, as we saw in the outcomes of the RCT. And as we have previously shown, the trained IPs showed better guideline adherence than the IPs in the control group [13].

The changes observed in the behavioural determinants appeared to last for a period of at least 3 months after the training took place. The fact that the control group showed a decrease in self-efficacy and in knowledge and skills indicates that there was no stimulating effect as a result of the measurements themselves; in fact these behavioural determinants actually faded with time. Although we expected there to be associations between the changes in ASE determinants and the improvements in guideline adherence, this was only marginally confirmed in this study for the determinants knowledge and skills. It is however possible that our training programme did induce the changes in these ASE determinants, as well as the improvements in guideline adherence.

### Strengths and Limitations of This Study

A strength of this study was that the questionnaires were developed on the basis of a theoretical model (the ASE model), and that they proved to be sufficiently reliable, while the constructs of this model were adjusted for the specific context of insurance medicine. Another strength was the high response rate of the questionnaires (100 %), which was thanks to the design of the study. In this design there was a follow-up measurement after 3 months, giving the participants in the intervention group the opportunity to put into practice what they had learned and practised during the training programme. Finally, it was possible to link the ASE determinants to the main outcome of the RCT—guideline adherence—thereby providing insight into IPs’ behaviour towards guidelines.

A limitation of this study was the low number of participants (40) used. Another limitation might be the fact that we studied changes in separate ASE determinants of behaviour as a result of the implementation strategy, while we were previously not able to confirm the ASE model in a cross-sectional analysis. However, in the present study it was possible to link such changes in ASE determinants to improvements in the levels of guideline adherence. Although the relationships between the ASE determinants of IPs’ behaviour at baseline were not strong, ASE determinants did change after we changed the behaviour of IPs by training them in applying the guidelines for depression—in fact all four ASE determinants changed significantly in the expected direction in this group. An explanation for this phenomenon might be that the intervention directly influences all ASE determinants. An additional explanation could be that the ASE model is a better fit when describing changes in behaviour, instead of exploring behaviour only at a single point in time. However, the fact that we could only demonstrate changes in the separate ASE determinants, and not in the *relationships* between the ASE determinants as a result of the implementation strategy, might well be a methodological limitation.

In the present study, three different kinds of bias might have occurred, which might also be regarded as methodological limitations. Firstly, the IPs participated on a voluntary basis, which might have induced a selection bias. However, since both the intervention and control groups were vulnerable to this bias, it might have reduced the contrast between both groups with regard to outcomes. Secondly, a literature search that assessed trends in self-reported adherence of clinicians to practice guidelines demonstrated that self-reported adherence levels exceeded the objective levels, resulting in a median over-estimation of adherence of 27 % [18]. Potential overestimation of self-reported guideline adherence may also have occurred in our study, but could not negatively influence our results since this bias accounts for both groups. Finally, in the follow-up questionnaire the participants were asked to fill in items relating to the intervention. Since the intervention took place 3 months previously this made their answers vulnerable to recall bias—they might have forgotten relevant facts, or they might have interpreted facts differently. On the other hand, the three-month interval was needed in order for the participants of the intervention group to reflect on what they had learned in the training programme. Furthermore, during the three-month interval they had the opportunity to put the acquired knowledge and skills concerning the guidelines into practice.

### Comparison with Other Studies

Other studies have suggested that theoretical models such as the Theory of Planned Behaviour (TPB) and its derivative, the attitude, social norm, self-efficacy (ASE) model, could help to identify ways of improving physician adherence or even to predict that behaviour [19, 20]. Recently, one study reported that the ASE model appears to be suitable for the description of the assessment behaviour of IPs [21]. The results of our study confirm this. The insurance physicians who received the implementation strategy demonstrated not only a higher level of guideline adherence [13], but also significant improvements in the determinants of their behaviour. The insurance physicians in our study increased their attitude, self-efficacy and intention in applying the guidelines, all determinants that are precursors for the intended behaviour, i.e. use of guidelines. The determinant knowledge and skills increased far less than the determinants attitude, self-efficacy and intention in our study.

Physicians’ knowledge of guidelines alone however, seems not to lead to better guideline adherence, as others have also shown [22, 23]. Furthermore, multifaceted interventions such as our implementation strategy are known to improve attitudes and behaviour, while stand-alone teaching only improves knowledge [24]. A cross-sectional survey carried out among Flemish occupational health physicians showed that the majority of physicians had a positive attitude toward implementing guidelines, but the physicians mentioned barriers in legislative framework, education and information structure [25]. Given our positive results, the newly developed implementation strategy may well have been successful in removing such barriers in education and information structure. The combination of the educational strategies used in the training programme together with the translation of the guidelines into practical and useful tools for the IPs was probably what stimulated the IPs’ attitude, self-efficacy, knowledge and skills, and intentions regarding use of the guidelines for depression.

### Practical Implications

The implications of the newly developed implementation strategy, consisting of a multifaceted training programme, are encouraging. The training programme itself took only one day of the physicians’ time. Similar training programmes could also be developed for other guidelines. This programme suited the needs of the physicians and was linked to their daily practice through the use of realistic case histories on video, which simulated clinical practice and contained evidence-based medicine. The IPs were then able to apply this evidence-based medicine in daily practice and gain experience in applying the guidelines for depression. Implementation of guidelines was also facilitated by the use of various tools. Educational programmes aimed at improving guideline adherence should be aimed not only at gaining knowledge but also at practising skills.

## Conclusions

The newly developed implementation strategy significantly increased the levels of insurance physicians’ attitude, self-efficacy, intention, and knowledge and skills with regard to their use of the guidelines for depression. These changes in determinants of behaviour might indicate positive changes in IPs’ behaviour regarding their use of the guidelines for depression. The improvements were achieved following a multifaceted one-day training programme, and lasted for at least 3 months. Although the levels of IPs’ guideline adherence improved after receiving the implementation strategy, this gain could only be related to increased levels of knowledge and skills. Improving knowledge and skills seems to be weakly related to the improvements in observed guideline adherence.

## Electronic supplementary material

Below is the link to the electronic supplementary material.

Supplementary material 1 (DOC 43 kb)
