# Supplementary material for: Changes in Insurance Physicians’ Attitudes, Self-Efficacy, Intention, and Knowledge and Skills Regarding the Guidelines for Depression, Following an Implementation Strategy
Source: J Occup Rehabil. 2012 Jul 5;23(1):148–56. doi: 10.1007/s10926-012-9378-9 (PMC3563952; doi:10.1007/s10926-012-9378-9)
Supplement: Supplementary file 1 — Supplementary material 1 (DOC 43 kb) [file 10926_2012_9378_MOESM1_ESM.doc]

**Appendix – ASE determinants questionnaire**

**Attitude concerning the use of the guidelines for depression**

All items are scored with Likert Scale (1-5) 1 = strongly disagree, 5 = strongly agree, if not reported otherwise

| 1. The guidelines for depression can support the IP with making complicated decisions |
| --- |
| 2. The guidelines for depression stimulates professionalization of the IPs |
| 3. Working in concordance with the guidelines for depression is too rigid for the individual  client |
| 4. The guidelines for depression provide for an increase in quality of assessment |
| 5. The guidelines for depression can improve the relationship between IP and the client |
| 6. The guidelines for depression are a threat to the autonomy of the IP |
| 7. Working in concordance with the guidelines for depression hinders professionals in making them familiar with new insights concerning depression |
| 8. I agree with the content of the guidelines for depression |
| 9. My attitude towards the guidelines for depression is positive |

**Self-efficacy concerning the use of the guidelines for depression**

| 1. I feel sufficiently equipped for applying the guidelines for depression |
| --- |
| 2. The guidelines for depression positively influence the quality of my assessments in practice |
| 3. The guidelines for depression to me are useful for: |
| A. Getting my assessment structured |
| B. Taking away my doubts |
| C. Strengthening my process of taking decisions |
| D. Writing down my work disability report |
| E. Preparing my assessment interview |
| F. Freshening up my knowledge |
| 4. The information presented in the guidelines for depression to me is:  Too complex; Just right; Too simple, or Not known |
| 5. How do you think of the clarity of the following aspects of the guidelines for depression?  A. The aim of the guidelines, to me is: Not clear; A little bit clear; or Completely clear  B. Assessing the prognosis to me is: Not clear; A little bit clear; or Completely clear |

**Knowledge and Skills concerning the use of the guidelines for depression**

| 1. I have sufficient knowledge to apply the guidelines for depression |
| --- |
| 2. I have the skills to work in concordance with the guidelines for depression |
| 3. I feel needs for further training and exercising in the use of the guidelines for depression |
| 4. I am able to organize my work in order to apply the guidelines for depression |
| 5. Learning to work in concordance with the guidelines for depression takes more time from me, than I have at disposal |
| 6. I have difficulties to integrate the use of the guidelines for depression in my daily work routine |
| 7. Present (disability) legislation leaves enough room for working in concordance with the guidelines for depression |
| 8. I believe that applying the guidelines for depression is practically feasible |

**Intention to use the guidelines for depression**

| 1. I have the intention to use or keep using elements of the guidelines for depression |
| --- |
| 2. I expect to use elements from the guidelines for depression in the near future |
| 3. I am intending to use or keep using the complete guidelines for depression |
| 4. I think the guidelines for depression are useful for taking decisions concerning the assessment of the work limitations |
| 5. Working in concordance with the guidelines for depression should be compulsory |
| 6. The clients, which I assess, benefit from the implementation of the guidelines for depression |
| 7. Too my opinion, there are clients, for whom the guidelines for depression are not applicable |
| 8. The guidelines for depression probably will be used in appeal cases |
| 9. The guidelines for depression probably will contribute to a decrease in lost appeal cases |
| 10.The guidelines for depression will contribute to a higher uniformity in the work disability assessments of clients with depression |
